# Prevention of Radial Oxygen Loss Is Associated With Exodermal Suberin Along Adventitious Roots of Annual Wild Species of *Echinochloa*

**DOI:** 10.3389/fpls.2019.00254

**Published:** 2019-03-11

**Authors:** Masato Ejiri, Katsuhiro Shiono

**Affiliations:** Laboratory of Plant Ecophysiology, Graduate School of Bioscience and Biotechnology, Fukui Prefectural University, Eiheiji, Japan

**Keywords:** barrier to radial oxygen loss, Casparian strip, exodermis, hypoxia, lignin, suberin, waterlogging

## Abstract

Internal aeration is crucial for root growth under waterlogged conditions. Some wetland plants have a structural barrier that impedes oxygen leakage from the basal part of roots called a radial oxygen loss (ROL) barrier. The ROL barrier reduces loss of oxygen transported via the aerenchyma to the root tips, enabling root growth into anoxic soil. The roots of some plants develop an ROL barrier under waterlogged conditions, while they remain leaky to oxygen under well-drained or aerated conditions. The main components of the inducible ROL barrier are thought to be suberin and lignin deposited at the outer cellular space (apoplast) in the outer part of roots. On the other hand, a few wetland plants including a species of *Echinochloa* form a constitutive ROL barrier, i.e., it is formed even in the absence of waterlogging. However, little is known about the components of constitutive ROL barriers. An ROL barrier is considered to be a characteristic of wetland species because it has not been found in any non-wetland species so far. Here, we examined whether *Echinochloa* species from non-waterlogged fields also form an inducible or constitutive ROL barrier. We found that three species of *Echinochloa* from non-waterlogged fields constitutively developed an ROL barrier under aerated conditions. Over 85% of their root exodermis cells were covered with suberin lamellae and had well-developed Casparian strips. These substances inhibited the infiltration of an apoplastic tracer (periodic acid), suggesting that the ROL barrier can also prevent the entry of phytotoxic compounds from the soil. Unlike the other *Echinochloa* species, *E. oryzicola*, which mainly inhabits rice paddies, was found to lack a constitutive ROL barrier under aerated conditions. Although close to 90% of its sclerenchyma was well lignified, it leaked oxygen from the basal part of roots. A high percentage (55%) of the root exodermis cells were not fortified with suberin lamellae. These results suggest that suberin is an important component of constitutive ROL barriers.

## Introduction

*Echinochloa* is a grass with both annual and perennial species. The annual species are highly pernicious weeds in rice paddies (Rao et al., 2007; Kraehmer et al., 2016). Three such species are known: *Echinochloa crus-galli*, *E. colona*, and *E. oryzicola* (Table 1). They are well adapted to various soil water situations and their habitats range from waterlogged paddy fields to well-drained crop fields (Yamasue, 2001; Rao et al., 2007; Tanesaka et al., 2010). Their life cycles and morphological characters closely resemble those of rice (Barrett, 1983), which makes it difficult to remove them. Although most weeds cannot grow and survive in the waterlogged soil in rice paddies, some *Echinochloa* species have well adapted to and become dominant in these habitats (Kraehmer et al., 2016). A better understanding of how these species acclimate to waterlogging will help to develop more effective herbicides or crop cultivation methods for controlling them.

**Table 1:** Accessions of annual wild *Echinochloa* used in the present study.

Species	Conditions of seed	Region of	Chromosome	Accession
	collection site	origin	number^1-3^	
*Echinochloa crus-galli* var. *crus-galli*	Waterlogged field	Kyoto, Japan^∗^	2n = 6X = 54	ECC-WL
*E. crus-galli* var. *crus-galli*	Well-drained field	Sendai, Japan^∗^	2n = 6X = 54	ECC-D
*E. crus-galli* var. *formosensis*	Waterlogged field	Kyoto, Japan^∗^	2n = 6X = 54	ECF-WL
*E. crus-galli* var. *praticola*	Well-drained field	Sendai, Japan^∗^	2n = 6X = 54	ECP-D
*E. colona*	Well-drained field	Santa Cruz, Bolivia^∗∗^	2n = 6X = 54	EC-D
*E. oryzicola*	Waterlogged field	Kyoto, Japan^∗^	2n = 4X = 36	EO-WL


*Sources of seeds: ^∗^Prof. T. Yoshioka, Fukui Prefectural University; ^∗∗^Dr. Y. Nakayama, Osaka Prefecture University. ^*1,2*^Yabuno (1962, 1984); ^*3*^Aoki and Yamaguchi (2009).*

Under waterlogged conditions, plants can suffer from hypoxia or anoxia because the ability of oxygen do diffuse through the water to the soil is extremely low (Jackson et al., 1985). Other problems associated with waterlogging are the accumulation of phytotoxic compounds in the soil (Ponnamperuma, 1984; Ernst, 1990; Lamers et al., 1998; Kreuzwieser et al., 2004) and a decline in the availability of some nutrients (Laanbroek, 1990). The roots of wetland plants contain a large volume of aerenchyma, which provides a low-resistance pathway for diffusion of oxygen from the shoot to the root (Armstrong, 1979; Kawai et al., 1998; Mano et al., 2006; Shiono et al., 2011; Nishiuchi et al., 2012). Some wetland species also form a barrier to radial oxygen loss (ROL) (Colmer, 2003b). The ROL barrier forms at the basal part of roots, and reduces the loss of oxygen transported via the aerenchyma to the root tips. In the roots with an ROL barrier, oxygen at the root tips and short lateral roots can be maintained at a higher level to allow root elongation into hypoxic/anoxic soil (Armstrong and Armstrong, 2005). In some wetland plants including rice, an ROL barrier is induced by stagnant or waterlogged conditions, while a weak barrier or no barrier forms in well-drained or aerated conditions (Colmer et al., 1998, 2006; Visser et al., 2000; Colmer, 2003a; Garthwaite et al., 2003; Abiko et al., 2012). However, a few wetland plants including *Echinochloa* form a constitutive ROL barrier, i.e., it is formed even in the absence of waterlogging (Visser et al., 2000; McDonald et al., 2001, 2002; Manzur et al., 2015).

Suberin and lignin deposits in the apoplast (the outer cellular space) prevent movement of water, ions and mycorrhizal fungi through the apoplast and thus act as an apoplastc barrier (Aloni et al., 1998; Enstone et al., 2003). Suberin is a hydrophobic macromolecule built from long-chain fatty acids and glycerol (Enstone et al., 2003; Graca, 2015). Lignin is a complex of polyphenolic polymers (Barros et al., 2015). Casparian strips, which are present in radial and transverse cell walls in the early developmental stage (State I), are comprised of lignin and suberin (Zeier et al., 1999; Schreiber and Franke, 2011; Naseer et al., 2012). Suberin lamellae, which are deposited on the inner surface of cell walls and surround the symplast in the subsequent developmental stage (State II), are comprised of suberin (Zeier et al., 1999; Schreiber and Franke, 2011). Because suberin was observed to accumulate at the exodermis and lignin was observed to accumulate at the sclerenchyma when plants formed an ROL barrier, the barrier is thought to be formed by deposits of suberin and lignin in the outer part of the roots (Watanabe et al., 2013). Thus, the ROL barrier is also thought to act as an apoplastic barrier, not only to impede oxygen loss but also to block the entry of phytotoxins (e.g., reduced metal ions) from waterlogged soil (Armstrong, 1979; Colmer, 2003b; Cheng et al., 2012). In wetland species that have an inducible ROL barrier, suberin has been suggested to be a major component of the barrier (Kulichikhin et al., 2014; Shiono et al., 2014b; Watanabe et al., 2017). However, little is known about which compound is the main constituent in constitutive ROL barriers. In *Cyperus eragrostis*, which has a constitutive ROL barrier, blue autofluorescence, a sign of both suberin and lignin, was stronger at the outer part of roots than in other species that do not have an ROL barrier (Manzur et al., 2015). It remains unclear which compound is the main constituent in constitutive ROL barriers.

An ROL barrier is considered to be a characteristic of wetland species because it has not been found in any non-wetland species so far. An annual wild *Echinochloa* species (*E. crus-galli* var. *mitis*) forms a constitutive ROL barrier (McDonald et al., 2001, 2002). However, it is not known whether the other annual wild *Echinochloa* species, which are distributed in both waterlogged and well-drained fields, form a constitutive barrier, and if they do, what it consists of. Here, we examined each of the three known wild annual *Echinochloa* species for constitutive ROL barriers. In the species that formed an ROL barrier, we also examined their chemical composition.

## Materials and Methods

### Plant Materials

This study was conducted with seeds of annual *Echinochloa* species collected from wild habitats (well-drained or waterlogged fields) in Japan and Bolivia (Table 1). The species included *Echinochloa crus-galli* (var. *crus-galli*, var. *formosensis* and var. *praticola*), *E. colona* and *E. oryzicola* and ecotypes of *E. crus-galli* var. *crus-galli* (Table 1). The Japanese and Bolivian seeds were kindly provided by Prof. Toshihito Yoshioka (Fukui Prefectural University) and Dr. Yuichiro Nakayama (Osaka Prefecture University), respectively.

### Growth Conditions

Seeds were sterilized for 30 min in 0.6% (w/v) sodium hypochlorite, washed thoroughly with deionized water, and for imbibition, placed in Petri dishes (8.5 cm diameter) containing about 6 ml of deionized water (about 1 mm water-depth) at 28°C under light to stimulate germination. The plants were grown in a controlled-environment chamber under constant light to avoid effects of circadian rhythm on gene expression (24-h light, 28°C, relative humidity over 50%, photosynthetic photon flux density at 248.8 μmol m^-2^ s^-1^). For the next phase, a soft sponge was floated on a container (380 mm × 260 mm × 160 mm high) of aerated quarter-strength nutrient solution (Colmer, 2003a; Shiono et al., 2011). Vertical slits were cut into the edges of the sponge. Four days after imbibition, each plant was slid into the slit so that the roots were submerged and the shoot protruded through the sponge into the light. Eight days after imbibition, the solution was replaced with aerated full-strength nutrient solution. To evaluate the constitutive ROL barrier under aerated conditions, 10 days after imbibition, plants were transplanted into aerated nutrient solution in 5-L pots (120 mm × 180 mm × 250 mm high, four plants per pot) for an additional 13–15 days. In each pot, a rectangular 2-cm-thick piece of foam was placed on the solution and aluminum foil was placed on the top of the foam to keep the solution dark. Vertical cuts were made on the four sides of the foam to accommodate stems. Then four plants were transferred to each pot, sliding the stems into the cuts. In this way, the roots were kept in the dark. The nutrient solutions were renewed every 7 days.

To evaluate the inducible ROL barrier under stagnant conditions, 10 days after imbibition, plants were transplanted into stagnant deoxygenated nutrient solution in 5-L pots for 13–15 days. The stagnant solution was prepared by adding 0.1% (w/v) agar to the nutrient solution and boiling the solution to dissolve the agar. The low concentration of agar produced a viscous liquid rather than a gel. By preventing convective movements, the solution mimics the changes in gas composition found in waterlogged soils (e.g., decreased oxygen and increased ethylene) (Wiengweera et al., 1997). The solution was poured into the pots and deoxygenated by bubbling N_2_ gas through two air stones at a flow rate of about 2.2 L min^-1^ for 15 min per pot. The dissolved oxygen (DO) level was confirmed to be less than 1.0 mg L^-1^ by DO meter (SG6-ELK, Mettler Toledo, Greifensee, Switzerland).

### ROL Barrier Formation

Methylene blue, which turns blue when exposed to oxygen, was used to evaluate oxygen leakage from roots. A solution containing 0.1% (w/v) agar was prepared, and after cooling, methylene blue (Sigma-Aldrich, St. Louis, MO, United States) was added to a final concentration of 13 mg l^-1^. The blue solution containing oxidized dye was reduced by addition of 130 mg l^-1^ sodium dithionite (Na_2_S_2_O_4_) to make it colorless. The test solution was placed in a clear plastic box (220 mm × 35 mm × 300 mm high) and the plant was held with tape so that the root–shoot junction was 50 mm below the surface, and the remainder of the shoot was in the air. The roots were stained for 30–60 min at room temperature. When the roots formed an ROL barrier, their basal parts were colorless (Shiono et al., 2011). We also measured the lengths of roots with an ROL barrier. The percentage of roots that formed an ROL barrier in a plant was calculated as: [(number of roots that formed a barrier to ROL)/(total root number)] × 100.

Radial oxygen loss from adventitious roots range from 100 mm to 120 mm length without lateral roots (i.e., it seems to be relatively young and active roots) was measured with Pt cylindrical root-sleeving O_2_ electrodes (Armstrong and Wright, 1975; Armstrong, 1994). The plant was placed in clear plastic boxes (55 mm × 55 mm × 300 mm high) fitted with rubber lids. The boxes were filled with an O_2_-free medium containing 0.1% (w/v) agar, 0.5 mM CaSO_4_ and 5 mM KCl. The shoot base was fixed to the rubber lids, so that the shoot was in the air and the root was in the O_2_-free medium. An adventitious root was inserted through the cylindrical root-sleeving O_2_ electrode (internal diameter, 2.25 mm; height, 5.0 mm). For calculation of ROL, root diameters of the position were measured with a micrometer caliper. ROL was measured in a lighted room kept at a constant 23°C.

### Histochemical Staining

Adventitious roots (100–120 mm length) without lateral roots were cut at the root–shoot junction. Their basal parts (15–25 mm below root–shoot junction) were embedded in 5% (w/v) agar. Root cross-sections of ca. 100 μm thickness were made using a vibrating microtome (Leica VT1200S, Leica Biosystems, Wetzlar, Germany). The cross-sections were made transparent by incubating them in lactic acid saturated with chloral hydrate at 70°C for 60 min (Lux et al., 2005). To detect suberin lamellae in the basal parts, we used 0.01% (w/v) Fluorol Yellow 088 in polyethylene glycol 400 as described by Brundrett et al. (1991). Suberin lamellae were visualized as a yellowish-green fluorescence excited by UV light. The cross-sections were viewed with an 02 UV filter set, an Axio Imager.A2 and an AxioCam MRc CCD camera (all Carl Zeiss, Oberkochen, Germany). To visualize lignin, transparent cross-sections of the basal parts were stained for 3 min with saturated phloroglucinol in 20% (w/w) hydrochloric acid at room temperature (Jensen, 1962). The reagent reacts with cinnamyl aldehyde groups in the lignin to produce an orange/red color under white light. The cross-sections were viewed with the Axio Imager.A2 microscope and AxioCam MRc CCD camera. Casparian strips in the basal parts were stained with 0.1% (w/v) berberine hemisulfate and 0.5% (w/v) aniline blue (Brundrett et al., 1988), which appears as bright white fluorescence under UV light. The cross-sections were viewed with the 02 UV filter set, Axio Imager.A2 and AxioCam MRc CCD camera. The ratio of cells with suberin lamellae, lignin or Casparian strips was determined by manually counting the numbers of cells in each photograph. To reduce bias, we randomly selected 30 cells in a cross-section derived from four independent roots.

### Permeability Test

Adventitious roots (100–120 mm length) without lateral roots were cut at the root–shoot junction. The permeability of the exodermal layers at the basal parts (15–25 mm below root–shoot junction) was assessed with an apoplastic tracer, periodic acid (Soukup et al., 2002; Shiono et al., 2014a; Pecková et al., 2016). The cut ends were covered with lanolin (Sigma-Aldrich) to prevent penetration of tracer. The roots were incubated in 0.1% (w/v) periodic acid (H_5_IO_6_) (Sigma-Aldrich) for 1 h, washed thoroughly with deionized water, incubated in reducing solution [1 g of potassium iodide (Wako) and 1 g of sodium thiosulfate (Wako) dissolved in 50 ml of water and acidified with 0.2 ml of 5 M hydrochloric acid (Wako)] for 1 h at room temperature, washed thoroughly with deionized water and incubated overnight at 4°C in the dark. The basal parts (17.5–22.5 mm below root–shoot junction) of adventitious roots were embedded in 5% (w/v) agar and cut in ca. 100-μm-thick cross-sections with a vibrating microtome (Leica VT1200S, Leica Biosystems). The sections were stained with Schiff’s reagent (Sigma-Aldrich) for 2 min and washed twice with 75% (v/v) glycerol (Wako). Periodic acid that penetrated into root tissue was visualized as a purple color under white light with the above microscope and camera.

### Root Porosity

Root porosity is the ratio of the gas volume to the volume of roots:

$$P=\frac{V_{g}}{V_{r}}\times 100,$$

where *V_g_* is the gas volume in the roots (including aerenchyma and intercellular space) and *V_r_* is the volume of the root tissue. It is measured by determining root buoyancy before and after vacuum infiltration of water into the gas spaces in the roots (Katayama, 1961; Raskin, 1983; Thomson et al., 1990).

Adventitious roots were separated from the shoot and cut into 50 mm segments. All of the segments from one plant were combined, gently blotted to remove excess water and weighed (*w1*). Three paper clips were used to hold the segments together and act as a sinker. Using an underhook balance (PA213CJP, Ohaus Corporation, Parsippany, NJ, United States), which weighs objects suspended below the balance, we measured the weight of just the paper clips hanging from a metal hook below the balance in a 2-L beaker of water (*w2*) and the weight of the roots held with the same paper clips in the water (*w3*). A smaller beaker containing the roots and paper clips in the water was placed in a vacuum desiccator and subjected to two 15-min periods of light vacuum (pressure, -50 kPa) to release the gas in the roots and infiltrate the roots with water. Finally, the paper clips and infiltrated roots were weighed in water (*w4*). Following Thomson et al. (1990), the above equation can then be expressed as

$$P=\frac{w4-w3}{w1+w2-w3}\times 100$$

### Growth Parameters

Plants were harvested after 14 days treatment in aerated or stagnant deoxygenated nutrient solutions. Leaf age, shoot length, the numbers of roots and the longest root length were recorded per plant. Shoots and roots were dried in an oven at 60°C for 3 days and weighed. For each growth parameter, percent control was calculated as: [(value under stagnant conditions)/(average value under aerated conditions)] × 100.

### Statistical Analysis

Means of root porosity and growth parameters among *Echinochloa* accessions were compared with one-way ANOVA and Tukey HSD for multiple comparisons at the 5% probability level. Means of root porosity and growth parameters between aerated and stagnant conditions were compared with a two-sample *t*-test. The data were analyzed with SPSS 16.0 for Windows (SPSS Inc., Chicago, IL, United States). The percentage of roots that formed an ROL barrier in a plant and the ratios of cells with suberin lamellae, lignin or Casparian strips were compared with Fisher’s exact test at the 5% probability level. The data were analyzed with R version 3.5.0 (R Core Team, 2018) (R packages: pwr).

## Results

### All but One Species of *Echinochloa* Formed a Constitutive Barrier to ROL

**FIGURE 1:**
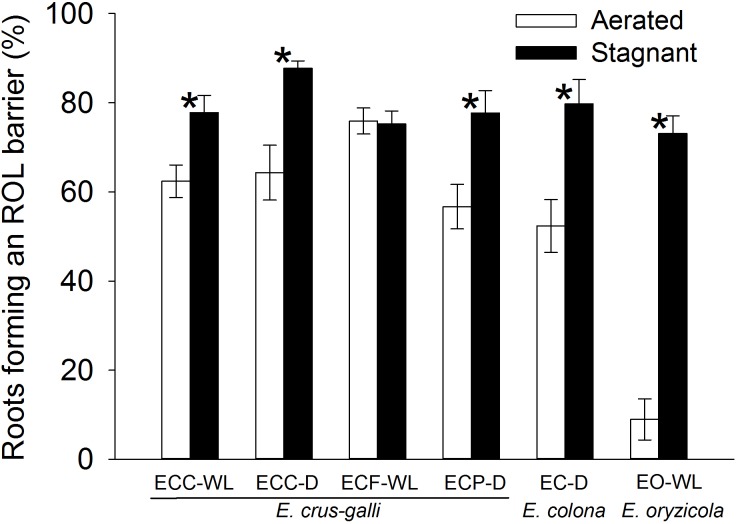
Percentages of the roots that formed an ROL barrier in *Echinochloa* accessions grown under aerated or stagnant conditions for 14 days. Methylene blue was used to evaluate the formation of ROL barrier in roots. Means ± SE. *n* = 5 or 6. Asterisks denote a significant difference between aerated and stagnant conditions (*P* < 0.05, Fisher’s exact test). Plants were grown in aerated nutrient solution for 10 days, and then transferred to deoxygenated stagnant 0.1% agar solution or continued in aerated nutrient solution for 14 days. Abbreviations (collected from): ECC-WL, *E. crus-galli* var. *crus-galli* (waterlogged field); ECC-D, *E. crus-galli* var. *crus-galli* (well-drained field); ECF-WL, *E. crus-galli* var. *formosensis* (waterlogged field); ECP-D, *E. crus-galli* var. *praticola* (well-drained field); EO-WL, *E. oryzicola* (waterlogged field); EC-D, *E. colona* (well-drained field).

**FIGURE 2:**
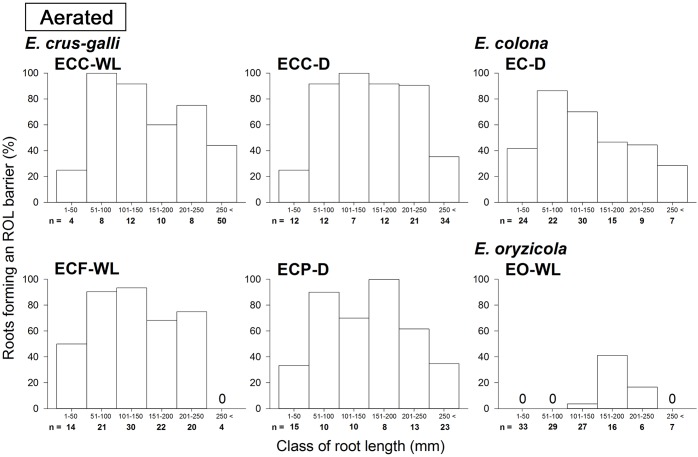
Distributions of root length of adventitious roots with an ROL barrier in *Echinochloa* accessions under aerated conditions. The data in this figure are replotted from the data in Figure 1 based on root length. All roots in five or six plants were used for each accession. Methylene blue was used to evaluate the formation of an ROL barrier in roots. Plants were grown in aerated nutrient solution for 10 days, and continued in aerated nutrient solution for 14 days.

**FIGURE 3:**
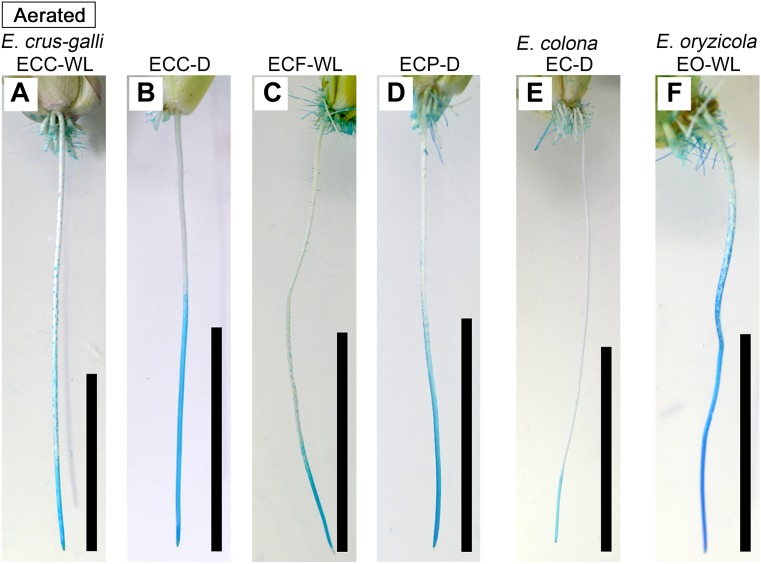
Patterns of oxygen leakage from adventitious roots in *Echinochloa* accessions under aerated conditions. **(A–D)** *E. crus-galli*. **(E)** *E. colona*. **(F)** *E. oryzicola*. Oxygen leakage from adventitious roots (100–120 mm length) was visualized with methylene blue. When the roots formed an ROL barrier, the basal part of roots was colorless. Blue color indicates that the methylene blue is oxidized by oxygen leaking from adventitious root. Plants were grown in aerated nutrient solution for 10 days, and then continued in aerated solution for 14 days. Scale bars: 50 mm.

In *E. colona* and all three varieties of *E. crus-galli*, 52–75% of the roots formed an ROL barrier under aerated conditions (Figure 1). However, in *E. oryzicola*, only 9% of the roots had an ROL barrier under aerated conditions. When these *Echinochloa* accessions including *E. oryzicola* were grown in stagnant solutions for 14 days, the percentage of roots that formed an ROL barrier ranged from 73 to 87%, values which were significantly higher than those in aerated conditions (*P* < 0.05, Figure 1), with the exception of ECC-WL. To clarify the difference between *E. oryzicola* and the other accessions, we evaluated the distributions of root lengths of adventitious roots with an ROL barrier formation under aerated conditions (Figure 2). In *E. colona* and all three varieties of *E. crus-galli*, most of the adventitious roots over 51 mm-length formed an ROL barrier (Figure 2) and none of their basal parts stained blue (Figure 3A–E and Supplementary Figures 1A–E). However, blue-stained spots were occasionally observed at the root surface where lateral roots were predicted to emerge (Supplementary Figures 1A–D).

On the other hand, in *E. oryzicola* grown under aerated conditions, roots shorter than 150 mm-length rarely formed an ROL barrier (Figure 2, EO-WL). Interestingly, slit-like stained spots were observed along the basal part of the roots (Figure 3F and Supplementary Figure 1F). These roots were frequently shorter than 150 mm (Supplementary Figure 2, EO-WL). When *E. oryzicola* was grown in stagnant solutions for 14 days, the adventitious roots did not stain blue (Supplementary Figure 3F) and none were observed with slit-like stained spots. This staining pattern was similar to the other *Echinochloa* accessions under aerated (Figure 3A–E) and stagnant conditions (Supplementary Figures 3A–E). Over 40% of the roots, even those shorter than 50 mm, formed an ROL barrier (Supplementary Figure 4, EO-WL).

**FIGURE 4:**
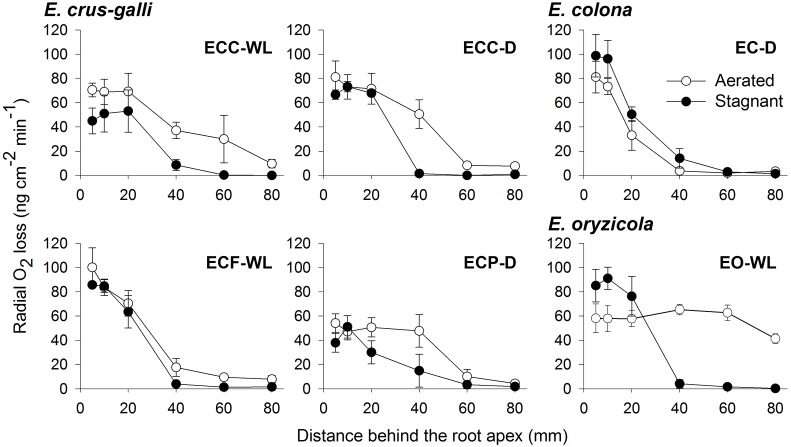
Rates of radial oxygen loss (ROL) along adventitious roots in *Echinochloa* accessions under aerated or stagnant conditions. ROL along adventitious roots (100–120 mm length) was measured by Pt cylindrical electrode. Means ± SE. *n* = 3 or 4. Plants were grown in aerated nutrient solution for 10 days, and then transferred to deoxygenated stagnant 0.1% agar solution or continued in aerated solution for 13–15 days.

To confirm the methylene blue results, oxygen leakage along 100–120 mm-long adventitious roots was also measured with an oxygen electrode. Under aerated conditions, *E. colona* and all three varieties of *E. crus-galli* constitutively formed a barrier to ROL along their adventitious roots (Figure 4). Both ecotypes of *E. crus-galli* var. *crus-galli* (ECC-WL and ECC-D) had a barrier to ROL along their adventitious roots. In each of the above accessions, ROL was substantially lower in the basal regions than in the more apical positions, consistent with the formation of an ROL barrier (Figure 4). The rate of ROL at the basal regions at 80 mm behind the root apex was extremely low (3.4–9.7 nmol O_2_ m^-2^ s^-1^). Interestingly, the adventitious roots in *E. oryzicola* (EO-WL) did not have an ROL barrier. The oxygen flux from the basal part to the root tips remained high (Figure 4). Moreover, the rate of ROL was 41.4 ± 3.9 nmol O_2_ m^-2^ s^-1^ at 80 mm behind the root apex, which was still higher than the rates in the other wild *Echinochloa* accessions. The higher ROL from the basal part of roots agreed with the visualized oxygen leakage pattern in *E. oryzicola* (Figure 3F and Supplementary Figure 1F). Under stagnant conditions, these *Echinochloa* accessions including *E. oryzicola* formed a barrier to ROL along their adventitious roots (Figure 4). Even in *E. oryzicola*, the rate of ROL at the basal regions at 60–80 mm from the root apex was extremely low (0–1.8 nmol O_2_ m^-2^ s^-1^). Both the methylene blue and oxygen electrode results showed that *E. colona* and all three varieties of *E. crus-galli* constitutively formed a constitutive ROL barrier under aerated conditions, but *E. oryzicola*, whose main habitat is waterlogged rice paddies, did not. The ability to form a constitutive ROL barrier was different among these accessions, but it was not related to the habitat (waterlogged or well-drained field).

In each of the *Echinochloa* accessions, the root porosity was high (over 10%) under aerated nutrient solutions (Table 2). The root porosity of the waterlogged ecotype *E. crus-galli* var. *crus-galli* (ECC-WL) was slightly higher than that of the well-drained ecotype (ECC-D), but the difference was not significant (*P* > 0.05). The porosity was slightly lower in the accessions from waterlogged fields [i.e., *E. crus-galli* var. *formosensis* (ECF-WL) and *E. oryzicola* (EO-WL)], than in the other accessions, but the difference was not significant. Although these accessions constitutively formed air spaces in their roots under aerated conditions, root porosity did not seem to be related to their habitat. For most of the accessions, both total root number and root porosity were greater under stagnant conditions than under aerated conditions (Table 2). In addition, root DW increased dramatically (2.5- to 6.5-fold) under stagnant conditions (Table 2). The longest root lengths in the accessions from well-drained fields (ECC-D, ECP-D, and EC-D) were reduced under the stagnant conditions, but those of the accessions from waterlogged fields (ECF-WL and EO-WL) were not reduced significantly (*P* > 0.05), except for ECC-WL. Other parameters (leaf age, shoot length and shoot DW) in almost all of the accessions were not significantly suppressed under stagnant conditions. The percent control values (stagnant/aerated) of both shoot DW and root DW in the accession from a waterlogged field (ECC-WL) were significantly higher than those in the accession from a well-drained field (ECC-D) (Table 2). However, root porosity, total root number, longest root length, shoot length and leaf age were not significantly different between the two ecotypes.

**Table 2:** Plant growth and root porosity in *Echinochloa* accessions under aerated or stagnant conditions.

Accessions	Aerated	Stagnant	*t*-test	% control
				(stagnant/aerated) ^§^
**Leaf age**		
ECC-WL	6.5 ± 0.1^bc^	6.8 ± 0.2^bc^	n.s.	103 ± 3^ab^
ECC-D	4.5 ± 0.2^d^	5.7 ± 0.5^c^	n.s.	126 ± 11^a^
ECF-WL	9.3 ± 0.2^a^	8.6 ± 0.3^a^	n.s.	92 ± 3^b^
ECP-^d^	7.0 ± 0.3^b^	7.2 ± 0.2^b^	n.s.	103 ± 3^ab^
EC-^d^	5.7 ± 0.3^c^	5.9 ± 0.2^c^	n.s.	103 ± 3^ab^
EO-WL	7.0 ± 0.2^b^	6.4 ± 0.0^bc^	n.s.	92 ± 0^b^
**Shoot length (cm)**		
ECC-WL	41.6 ± 0.8^bc^	43.5 ± 0.6^b^	n.s.	105 ± 2^ab^
ECC-^d^	39.5 ± 3.4^bc^	46.0 ± 0.9^ab^	n.s.	116 ± 2^a^
ECF-WL	59.5 ± 3.1^a^	52.9 ± 1.6^a^	n.s.	89 ± 3^b^
ECP-^d^	47.3 ± 6.5^ab^	47.0 ± 1.9^ab^	n.s.	99 ± 4^ab^
EC-^d^	30.5 ± 2.8^c^	27.4 ± 2.5^c^	n.s.	90 ± 8^b^
EO-WL	44.3 ± 1.0^abc^	41.5 ± 1.0^b^	n.s.	94 ± 2^b^
**Longest root length (cm)**		
ECC-WL	43.5 ± 1.8^a^	24.0 ± 1.3^a^	^∗^	55 ± 3^c^
ECC-^d^	45.5 ± 2.9^a^	24.0 ± 0.4^a^	^∗^	53 ± 1^c^
ECF-WL	22.8 ± 2.3^c^	27.1 ± 0.9^a^	n.s.	119 ± 4^a^
ECP-^d^	34.6 ± 3.7^ab^	24.6 ± 0.9^a^	^∗^	71 ± 3^c^
EC-^d^	26.3 ± 2.5^bc^	14.9 ± 2.1^b^	^∗^	57 ± 8^c^
EO-WL	28.5 ± 1.4^bc^	26.1 ± 1.4^a^	n.s.	92 ± 5^b^
**Total root number**		
ECC-WL	17 ± 1^b^	37 ± 5^bc^	^∗^	216 ± 30^a^
ECC-^d^	24 ± 2^b^	33 ± 2^bc^	^∗^	138 ± 8^ab^
ECF-WL	44 ± 6^a^	63 ± 2^a^	^∗^	143 ± 5^ab^
ECP-^d^	21 ± 1^b^	36 ± 4^bc^	^∗^	176 ± 16^ab^
EC-^d^	19 ± 3^b^	25 ± 6^c^	n.s.	135 ± 31^ab^
EO-WL	42 ± 4^a^	44 ± 2^b^	n.s.	105 ± 4^b^
**Shoot DW (g)**		
ECC-WL	0.37 ± 0.02^bc^	0.97 ± 0.08^ab^	^∗^	266 ± 22^a^
ECC-^d^	0.63 ± 0.11^abc^	0.84 ± 0.04^b^	n.s.	134 ± 7^b^
ECF-WL	1.19 ± 0.26^a^	1.21 ± 0.06^a^	n.s.	101 ± 5^b^
ECP-^d^	0.80 ± 0.17^ab^	0.98 ± 0.05^ab^	n.s.	123 ± 6^b^
EC-^d^	0.13 ± 0.03^c^	0.23 ± 0.07^c^	n.s.	178 ± 56^ab^
EO-WL	0.75 ± 0.04^ab^	0.78 ± 0.05^b^	n.s.	103 ± 7^b^
**Root DW (g)**		
ECC-WL	0.08 ± 0.01^bc^	0.54 ± 0.02^a^	^∗^	650 ± 29^a^
ECC-^d^	0.13 ± 0.03a^bc^	0.44 ± 0.04^a^	^∗^	337 ± 31^b^
ECF-WL	0.21 ± 0.05^a^	0.63 ± 0.02^a^	^∗^	301 ± 11^b^
ECP-^d^	0.13 ± 0.02^abc^	0.52 ± 0.07^a^	^∗^	388 ± 49^ab^
EC-^d^	0.04 ± 0.01^c^	0.11 ± 0.05^b^	n.s.	302 ± 124^b^
EO-WL	0.17 ± 0.01^ab^	0.43 ± 0.06^a^	^∗^	251 ± 34^b^
**Root porosity (%)**		
ECC-WL	22.7 ± 1.8^a^	30.8 ± 0.6^a^	^∗^	135 ± 3^b^
ECC-^d^	18.9 ± 0.9^ab^	24.1 ± 0.4^a^	^∗^	127 ± 2^b^
ECF-WL	12.4 ± 1.7^c^	30.6 ± 0.9^a^	^∗^	246 ± 7^a^
ECP-^d^	15.8 ± 0.6^bc^	29.6 ± 1.2^a^	^∗^	188 ± 7^ab^
EC-^d^	17.2 ± 2.2^abc^	28.1 ± 6.2^a^	n.s.	164 ± 36^b^
EO-WL	11.9 ± 0.4^c^	28.9 ± 0.6^a^	^∗^	244 ± 5^a^


*The aerated and stagnant growth experiments were conducted simultaneously. Mean ± SE. *n* = 3 or 4. Different lower-case letters denote significant differences among wild *Echinochloa* accessions (*P* < 0.05, one-way ANOVA and then Tukey HSD for multiple comparisons). Asterisks indicate significant differences between means of aerated and stagnant conditions (*P* < 0.05, two-sample *t*-test) n.s., not significant. ^§^ % controls for each growth parameter were calculated as: [(value under stagnant conditions)/(average value under aerated conditions)] × 100. Plants were grown in aerated nutrient solution for 10 days, and then transferred to deoxygenated stagnant 0.1% agar solution or continued in aerated solution for 14 days.*

### Suberized Exodermis Was Found in the Roots Forming a Constitutive ROL Barrier

**FIGURE 5:**
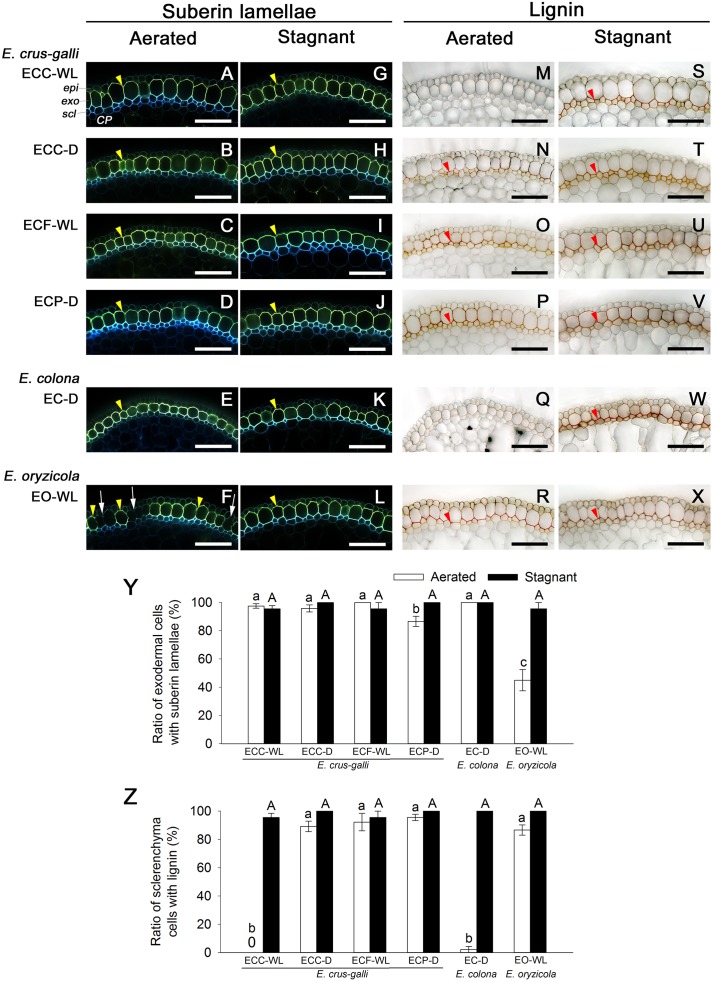
Suberization and lignification in the outer part of roots in *Echinochloa* accessions under aerated or stagnant conditions. Suberin lamellae and lignin deposits were observed in the basal parts (15–25 mm below root–shoot junction) of adventitious roots of 100–120 mm length. **(A–L)** Suberin lamellae at the exodermis. Suberin lamellae are indicated as yellow-green fluorescence with Fluorol Yellow 088 (yellow arrowhead). White arrows denote the site of exodermis/hypodermis without suberin lamellae. Blue fluorescence indicates autofluorescence. **(M–X)** Lignin deposits at the sclerenchyma. Lignin is indicated as orange/red with phloroglucinol-HCl (red arrowhead). *CP*, cortical parenchyma; *epi*, epidermis; *exo*, exodermis; *scl*, sclerenchyma. Scale bars: 100 μm. **(Y)** Ratios of cell numbers observed suberin lamellae. **(Z)** Ratios of cell numbers observed lignin. Means ± SE. *n* = 4. Different lower-case letters denote significant differences among *Echinochloa* accessions (*P* < 0.05, Fisher’s exact test for multiple comparisons). Plants were grown in aerated nutrient solution for 10 days, and then transferred to deoxygenated stagnant 0.1% agar solution or continued in aerated solution for 14 days.

In *E. colona* and all three varieties of *E. crus-galli* that formed a constitutive ROL barrier under aerated conditions, the basal parts (15–25 mm below root–shoot junction) of the adventitious roots (100–120 mm length) were clearly surrounded by well suberized exodermis, as shown by the yellowish-green fluorescence (Figure 5A–E). Over 85% of their exodermal cells clearly developed suberin lamellae (Figure 5Y). However, part of the exodermis of *E. oryzicola* (a species that does not form a constitutive ROL barrier) lacked the yellowish-green fluorescence of suberin lamellae (Figure 5F). Passage cells, which are a type of exodermal cell that lacks suberin lamellae, were observed frequently (Figure 5F), so that the ratio of cells with suberin lamellae (45%) was significantly lower than the ratios of the other accessions (Figure 5Y, *P* < 0.05). On the other hand, orange/red (from staining of lignin) at the sclerenchyma was observed at the basal part of roots in *E. oryzicola* (Figure 5R); 87% of the sclerenchyma cells developed lignin deposits (Figure 5Z). Three of four accessions in *E. crus-galli* (ECC-D, ECF-WL and ECP-D) had well lignified sclerenchyma (Figure 5N–P,Z). Lignin deposits were not observed at the sclerenchyma in *E. crus-galli* var. *crus-galli* (ECC-WL) or *E. colona* (EC-D) (Figure 5M,Q,Z), although both accessions constitutively formed an ROL barrier. Not all the accessions that formed an ROL barrier had well-lignified sclerenchyma, but development of a constitutive ROL barrier was associated with the presence of suberized exodermis surrounding the roots.

Under stagnant conditions, the basal parts of the adventitious roots of in all wild *Echinochloa* accessions including *E. oryzicola*, were clearly surrounded by well suberized exodermis (Figure 5G–L). *E. oryzicola*, which inducibly formed an ROL barrier under stagnant conditions, had hardly any passage cells (Figure 5L) and over 97% of their exodermal cells clearly developed suberin lamellae (Figure 5Y). Lignin deposits at the sclerenchyma were also observed in all of the *Echinochloa* accessions (Figure 5S–X). In both *E. crus-galli* var. *crus-galli* (ECC-WL) and *E. colona* (EC-D), over 96% of the sclerenchyma cells developed lignin deposits (Figure 5Z). Development of an inducible ROL barrier in *E. oryzicola* was also associated with lignification at the exodermis.

### Association of an Apoplastic Barrier With a Constitutive ROL Barrier

**FIGURE 6:**
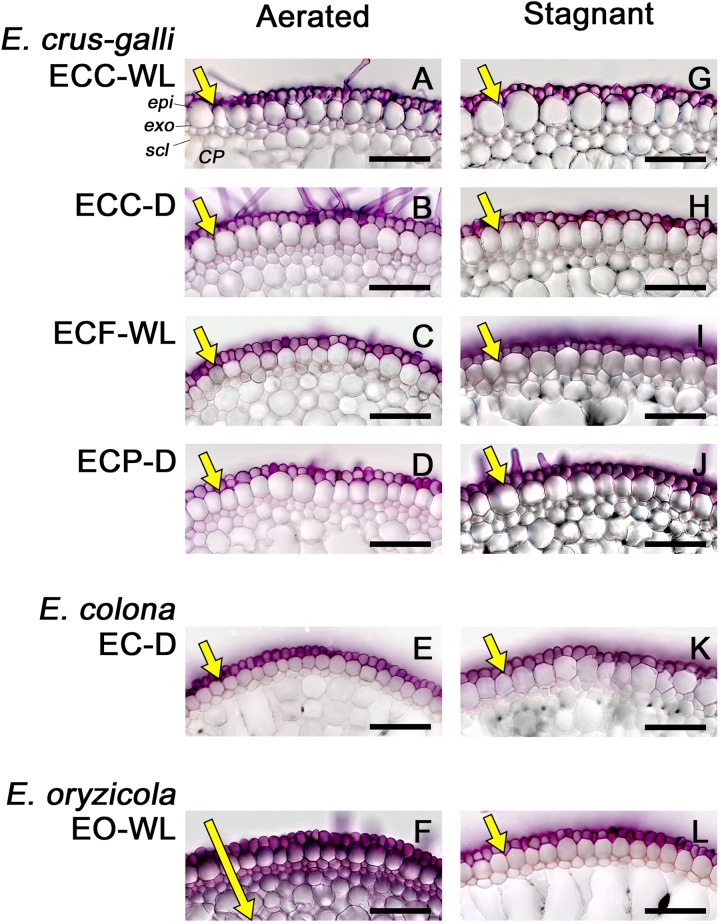
Permeability of the exodermis to an apoplastic tracer (periodic acid) in *Echinochloa* accessions under aerated or stagnant conditions. **(A–F)** Aerated conditions. **(G–L)** Stagnant conditions. The permeability of the exodermis was evaluated at the basal parts (17.5–22.5 mm below root–shoot junction) of adventitious roots of 100–120 mm length. Purple color indicates that periodic acid penetrated into root tissues. The length of yellow arrows indicates the extent of penetration. Plants were grown in aerated nutrient solution for 10 days, and then transferred to deoxygenated stagnant 0.1% agar solution or continued in aerated solution for 14 days. *CP*, cortical parenchyma; *epi*, epidermis; *exo*, exodermis; *scl*, sclerenchyma. Scale bars: 100 μm.

Because suberin lamellae help to form an apoplastic transport barrier that separates plant tissue from the surrounding conditions, we evaluated the ability of an apoplastic tracer (periodic acid) to penetrate the basal parts (15–25 mm below root–shoot junction) of 100–120 mm-long adventitious roots. In *E. colona* and all three varieties of *E. crus-galli* under aerated and stagnant conditions, the purple color of periodic acid was detected only in the epidermal cells (Figure 6A–E,G–K). Penetration of the tracer was blocked at the outside of the exodermis. In *E. oryzicola*, penetration of the tracer was also blocked at the outside of the exodermis under stagnant conditions (Figure 6L), but not under aerated conditions (Figure 6F). Thus, in all accessions, the basal part of roots that formed an ROL barrier developed an apoplastic barrier at the exodermis.

**FIGURE 7:**
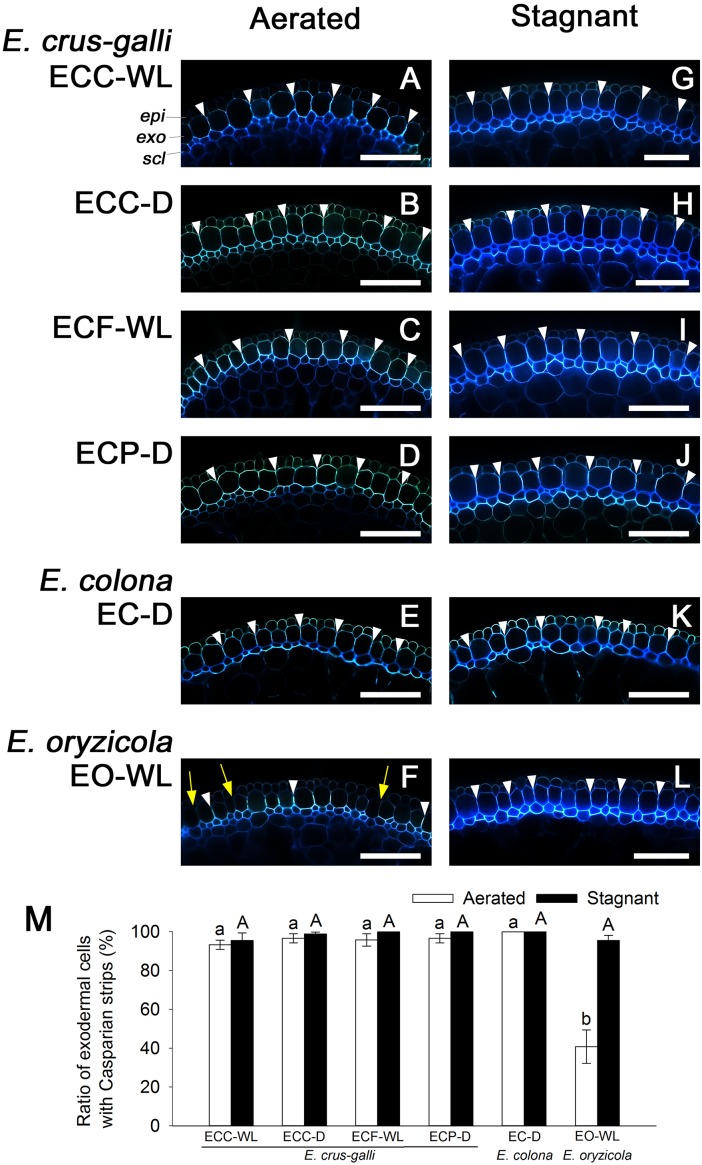
Casparian strips at the exodermis in *Echinochloa* accessions that were grown under aerated or stagnant conditions. Casparian strips (stained by berberine-aniline blue) were observed in the basal parts (15–25 mm below root–shoot junction) of adventitious roots of 100–120 mm length. **(A–L)** Casparian strips at the exodermis. Casparian strips are indicated as a bright white fluorescence with berberine-aniline blue. Representative Casparian strips are shown with white arrowheads. Yellow arrows denote the region of exodermis/hypodermis without Casparian strips. Blue fluorescence indicates autofluorescence. *CP*, cortical parenchyma; *epi*, epidermis; *exo*, exodermis; *scl*, sclerenchyma. Scale bars: 100 μm. **(M)** Ratios of cell numbers observed Casparian strips. Means ± SE. *n* = 4. Different lower-case letters denote significant differences among *Echinochloa* accessions (*P* < 0.05, Fisher’s exact test for multiple comparisons). Plants were grown in aerated nutrient solution for 10 days, and then transferred to deoxygenated stagnant 0.1% agar solution or continued in aerated solution for 14 days.

Casparian strips as well as suberin lamellae inhibit apoplastic transport at the exodermis. Like the suberin lamellae, Casparian strips were well developed in *E. colona* and all three varieties of *E. crus-galli* under aerated conditions (Figure 7A–E and Supplementary Figures 5A–E), but they were only patchy in *E. oryzicola* (Figure 7F and Supplementary Figure 5F). The number of cells that formed Casparian strips at the exodermis was also drastically lower in *E. oryzicola* than in the other *Echinochloa* accessions (*P* < 0.05) (Figure 7M and Supplementary Figure 5M). Under stagnant conditions, Casparian strips were clearly visible at the exodermis in *E. oryzicola* (Figure 7L and Supplementary Figure 5L) as well as the other *Echinochloa* accessions (Figure 7G–K and Supplementary Figures 5G–K). The number of cells that formed Casparian strips had risen to over 95% in *E. oryzicola* (Figure 7M and Supplementary Figure 5M). The development of Casparian strips at the exodermis was closely associated with the development of three structures: suberin lamellae, an apoplastic barrier against penetration of periodic acid and a constitutive ROL barrier.

## Discussion

A constitutive ROL barrier has been found in a limited number of wetland plants including one species of *Echinochloa* (*E. crus-galli* var. *mitis*) (McDonald et al., 2001, 2002). Here, by investigating all known annual wild *Echinochloa* species, we showed that *E. crus-galli* and *E. colona* had a constitutive ROL barrier under aerated conditions, but *E. oryzicola* did not (Figure 1, 4). Although *E. crus-galli* has several varieties and ecotypes, each of these varieties and ecotypes, including those previously investigated by McDonald et al. (2001, 2002), formed a constitutive ROL barrier. Under aerated conditions, *E. colona* and all accessions of *E. crus-galli* developed suberin lamellae (Figure 5A–E,Y) and Casparian strips (Figure 7A–E,M and Supplementary Figures 5A–E,M) at the exodermis, but *E. crus-galli* var. *crus-galli* (ECC-WL in Figure 5M) and *E. colona* (EC-D in Figure 5Q) did not develop lignified sclerenchyma. These results are consistent with the reductions of ROL from the basal part of roots (40–80 mm from the root apex) (Figure 4). Previous studies of the inducible ROL barrier in rice suggest that exodermal suberization is important for formation of an ROL barrier because genes and metabolites associated with suberin biosynthesis are strongly upregulated under ROL barrier induction, whereas genes associated with lignin biosynthesis were not (Kulichikhin et al., 2014; Shiono et al., 2014b). A *Zea nicaraguensis* chromosome segment introgression line in maize (IL#468) that formed an inducible ROL barrier also developed a well suberized exodermis along the basal parts of adventitious roots (Watanabe et al., 2017). However, a lignified epidermis was not observed (Watanabe et al., 2017). Accumulation of suberin at the exodermis was closely associated with a reduction of oxygen leakage at the basal parts of roots in *Tabernaemontana juruana*, an Amazonian tree species (De Simone et al., 2003) and *Phragmites australis* (Soukup et al., 2007), although it is not known whether these species form inducible or constitutive ROL barriers. *E. oryzicola* developed lignified sclerenchyma under aerated conditions (Figure 5R,Z) although this did not reduce oxygen leakage from basal part of roots (40–80 mm from the root apex) (EO-WL in Figure 4) and did not block the infiltration of an apoplastic tracer (Figure 6F). Although lignified sclerenchyma was not observed in *E. crus-galli* var. *crus-galli* and *E. colona* (Figure 5M,Q,Z), oxygen leakage from the basal part of the roots was impeded (ECC-WL and EC-D in Figure 4) and infiltration of the apoplastic tracer was blocked (Figure 6A,E). Additionally, when *E. oryzicola* formed an inducible ROL barrier under stagnant conditions (Figure 1, 4), its exodermis also had well suberized cells (Figure 5L,Y) and Casparian strips (Figure 7L,M and Supplementary Figures 5L,M). The *E. oryzicola* exodermis blocked the infiltration of the apoplastic tracer (Figure 6L). Lignin has roles in providing mechanical support and plant defense due to its resistance to degradation (Campbell and Sederoff, 1996; Schreiber et al., 1999; Barros et al., 2015). Some of the observed lignification at the sclerenchyma might provide mechanical support and plant defense, but the lignification does not appear to contribute to an ROL barrier. Like previous findings on inducible ROL barriers, our results suggest that suberin, but not lignin, is an important component of a constitutive ROL barriers.

### Oxygen Leakage Through Areas of Passage Cells (Windows)

Passage cells in the outer part of roots, which lack suberin lamellae, are found in some roots that are leaky to oxygen (Armstrong et al., 2000; Abiko et al., 2012). Areas of passage cells where oxygen leaks and lateral roots emerge have been called “windows” (Armstrong et al., 2000). Under aerated conditions, *E. oryzicola* does not develop an ROL barrier, as the basal part of its roots lost a substantial amount of oxygen (Figure 4). In addition, oxygen was found to leak from narrow slits in the basal part of roots (Figure 3F and Supplementary Figure 1). In *E. oryzicola* under aerated conditions, in agreement with its weak ROL barrier (Figure 4), most (55%) of its exodermal cells at the basal parts of roots were passage cells lacking suberin lamellae (Figure 5F,Y). Although the basal part of roots of maize species (including inbred line Mi29) grown in stagnant conditions have a suberized exodermis, they lose a substantial amount of oxygen, apparently because 4–15% of the exodermal cells are passage cells that lack suberin lamellae (Abiko et al., 2012). The *E. oryzicola* grown in aerated conditions had 4–14 times more passage cells than did Mi29, which appears to be the reason for the leakiness of its roots. Some of the other accessions with a constitutive barrier (i.e., with low ROL) also showed a few spots of methylene blue staining along the adventitious roots (Figure 3A–D and Supplementary Figure 1). These spots seemed to be the sites where lateral roots emerged (Supplementary Figure 1). Similarly, in *Phragmites australis*, oxygen was found to leak from areas of passage cells where the lateral root emerges (Armstrong et al., 2000). In agreement with the observations of Armstrong et al. (2000), the sites of lateral root emergence in *E. crus-galli* var. *crus-galli* (ECC-WL and ECC-D) and *E. crus-galli* var. *praticola* (ECP-D) under aerated conditions lacked suberin lamellae at the exodermis (Supplementary Figure 6). These observations support the idea that suberin lamellae impede the leakage of oxygen from the roots.

### Distribution of *Echinochloa* Species With an ROL Barrier

The ability to form a constitutive or inducible barrier is considered an adaptation to waterlogging (Colmer, 2003b). Two of the *E. crus-galli* accessions that came from waterlogged fields (ECC-WL and ECF-WL) formed an ROL barrier under aerated conditions (Figure 1, 4). However, *E. oryzicola* whose main habitat is waterlogged rice paddies (Yabuno, 1984; Yamasue, 2001), did not have a constitutive ROL barrier under aerated conditions (Figure 1, 4). When *E. oryzicola* was grown in stagnant conditions for 7 days, 40% of the roots formed an ROL barrier (EO-WL in Supplementary Figure 7). At 14 days after stagnant treatment, 73% of the roots formed an ROL barrier (EO-WL in Figure 1). Like rice, *E. oryzicola* had an inducible ROL barrier under stagnant conditions (EO-WL in Figure 1, 4 and Supplementary Figure 7). This might help *E. oryzicola* to adapt to waterlogged rice paddy fields.

So far, ROL barriers have been found only in wetland species (McDonald et al., 2002; Colmer, 2003b). However, some of the accessions from well-drained fields [two of *E. crus-galli* (ECC-D and ECP-D) and *E. colona* (EC-D)] constitutively formed an ROL barrier (Figure 1, 4). Moreover, these accessions had well developed apoplastic barriers at the exodermis (Figure 6B,D,E) with suberin (Figure 5B,D,E) and Casparian strips (Figure 7B,D,E and Supplementary Figures 5B,D,E). Our previous finding that a rice mutant (*reduced culm number1*) that lacks both suberin lamellae and Casparian strips did not block the infiltration of apoplastic tracers (periodic acid and berberine) (Shiono et al., 2014a), suggests that exodermal suberization and Casparian strips act as an apoplastic barrier. The apoplastic barrier at the exodermis serves to protect the plant from various environmental stresses, such as mycorrhizal infections (Damus et al., 1997), water loss to dry soil (Aloni et al., 1998) and penetration of ions (e.g., Na^+^) (Enstone et al., 2003; Ranathunge et al., 2011). The constitutive apoplastic barriers in these *Echinochloa* accessions collected from well-drained fields might help to adapt to waterlogging and other environmental stresses because they prepare the plants for environmental changes.

In most annual wild *Echinochloa* that form a constitutive ROL barrier, formation of the barrier was closely associated with exodermal suberization. A phylogenic analysis suggests that *E. crus-galli* (hexaploid) was derived from *E. oryzicola* (tetraploid) (Aoki and Yamaguchi, 2008, 2009). *E. oryzicola* is limited to waterlogged paddies, while *E. crus-galli* has several varieties that are adapted to wet and dry areas (Yamasue, 2001; Rao et al., 2007; Tanesaka et al., 2010). *E. crus-galli* may have diversified its habitat by acquiring a constitutive apoplastic barrier at the exodermis. Although it appears likely that a constitutive apoplastic barrier helps a plant to adapt to environmental stresses such as drought, high salinity and waterlogging, further studies using a larger number of wild accessions are needed to confirm this. *Echinochloa* appears to be well-suited for such studies because of its wide variety of species adapted to different environmental conditions. Such studies will lead to a better understanding of *Echinochloa*’s high adaptability to various environmental conditions and thus to develop better measures for their control.

## Author Contributions

ME and KS designed the experiments and wrote the draft of article. KS wrote the article and supervised the experiments. ME performed the most of the experiments and analyses.

## Conflict of Interest Statement

The authors declare that the research was conducted in the absence of any commercial or financial relationships that could be construed as a potential conflict of interest.
