# Supplementary material for: Prevention of Radial Oxygen Loss Is Associated With Exodermal Suberin Along Adventitious Roots of Annual Wild Species of Echinochloa
Source: Front Plant Sci. 2019 Mar 11;10:254. doi: 10.3389/fpls.2019.00254 (PMC6421306; doi:10.3389/fpls.2019.00254)
Supplement: Supplementary file 1 [file Data_Sheet_1.pdf]

Supplementary Material

Prevention of radial oxygen loss is associated with exodermal suberin along adventitious roots of annual wild species of *Echinochloa*

Masato Ejiri[1] and Katsuhiro Shiono[1, *]

*** Correspondence:** Katsuhiro Shiono: shionok@fpu.ac.jp

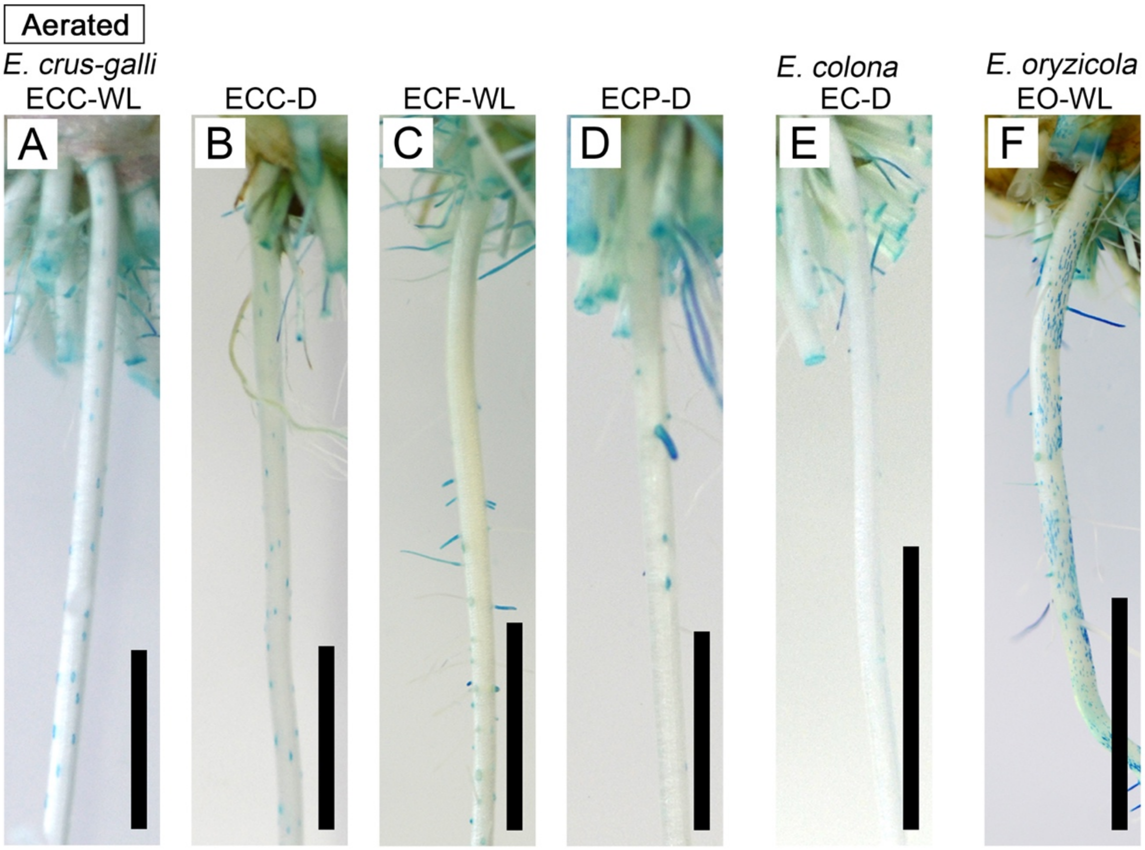

Supplementary Figure 1| Patterns of oxygen leakage from adventitious roots in *Echinochloa* accessions under aerated conditions. (A-D) *E. crus-galli.* **(E)** *E. colona.* **(F)** *E. oryzicola.* Oxygen leakage from adventitious roots (100-120 mm length) was visualized with methylene blue. When the roots formed an ROL barrier, the basal part of roots was colorless. Blue color indicates that the methylene blue is oxidized by oxygen leaking from adventitious root. Plants were grown in aerated nutrient solution for 10 days, and then continued in aerated solution for 14 days. Scale bars, 10 mm.

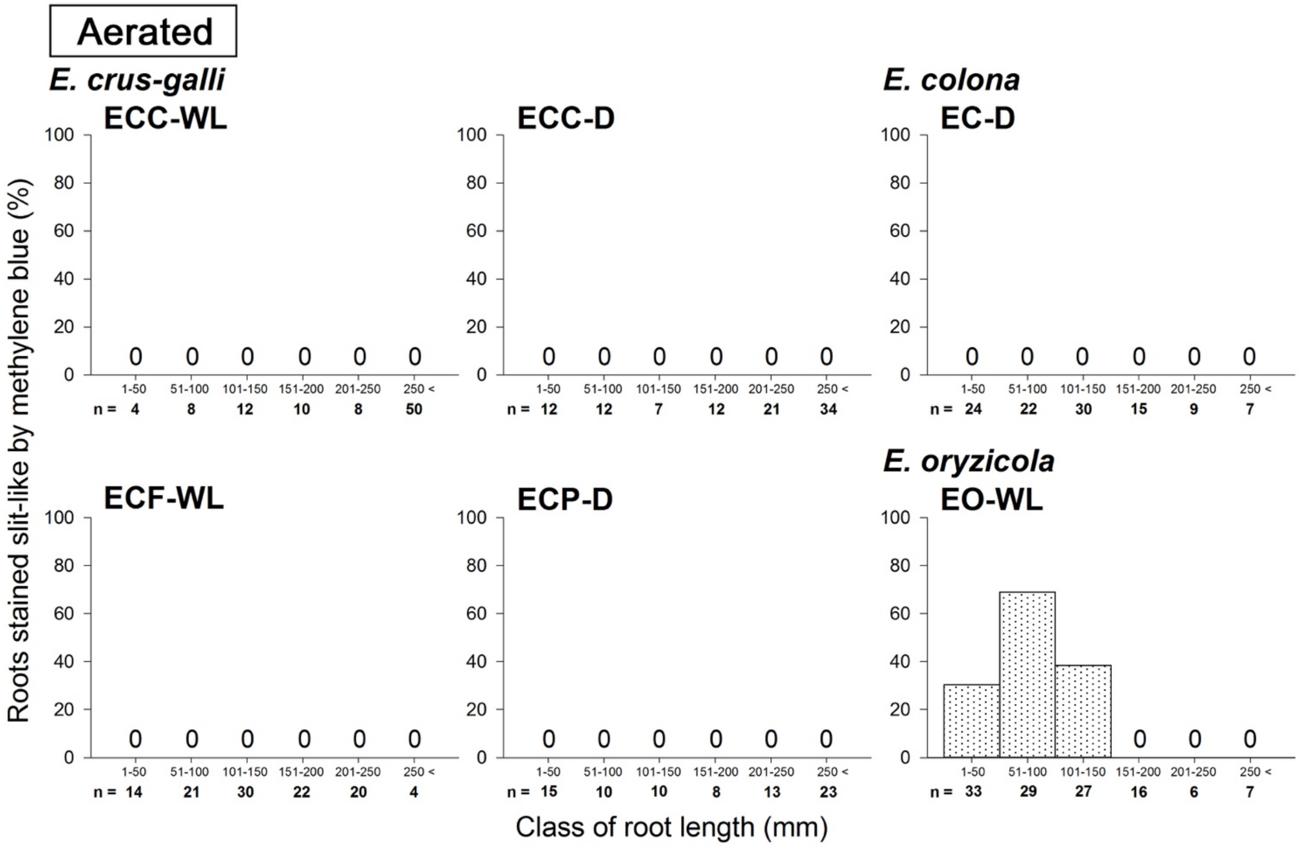

Supplementary Figure 2| Distributions of root length of adventitious roots with slit-like stained spots in *Echinochloa* accessions under aerated conditions. The slit-like stained spots along the basal part of roots (Figure 3F and Supplementary Figure 1F) were visualized by methylene blue staining. All roots in 5 or 6 plants were used for each accession. Plants were grown in aerated nutrient solution for 10 days, and continued in aerated nutrient solution for 14 days.

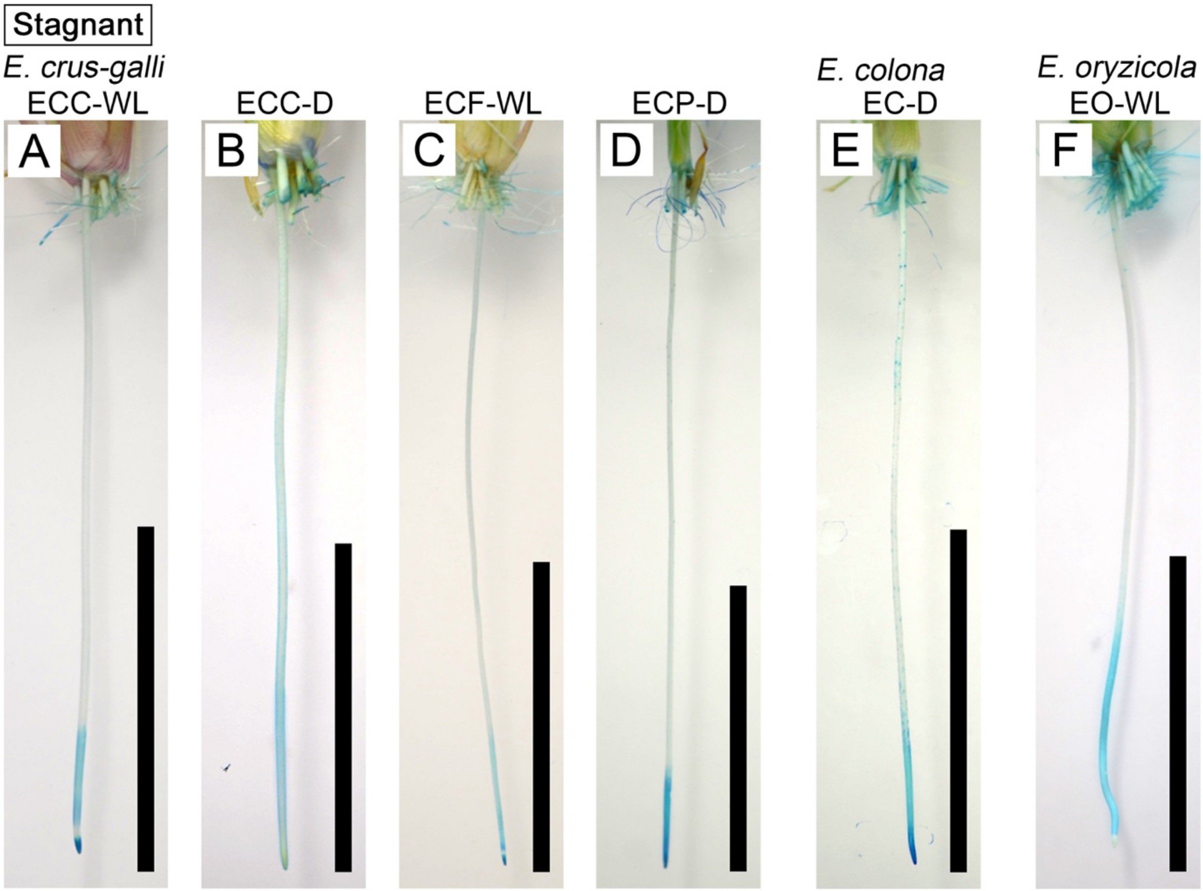

Supplementary Figure 3| Patterns of oxygen leakage from adventitious roots in *Echinochloa* accessions under stagnant conditions. (A-D) *E. crus-galli.* **(E)** *E. colona.* **(F)** *E. oryzicola.* Oxygen leakage from adventitious roots (100-120 mm length) was visualized with methylene blue. When the roots formed an ROL barrier, the basal part of roots was colorless. Blue color indicates that the methylene blue is oxidized by oxygen leaking from adventitious root. Plants were grown in aerated nutrient solution for 10 days, and then transferred to deoxygenated stagnant 0.1% agar solution for 14 days. Scale bars, 50 mm.

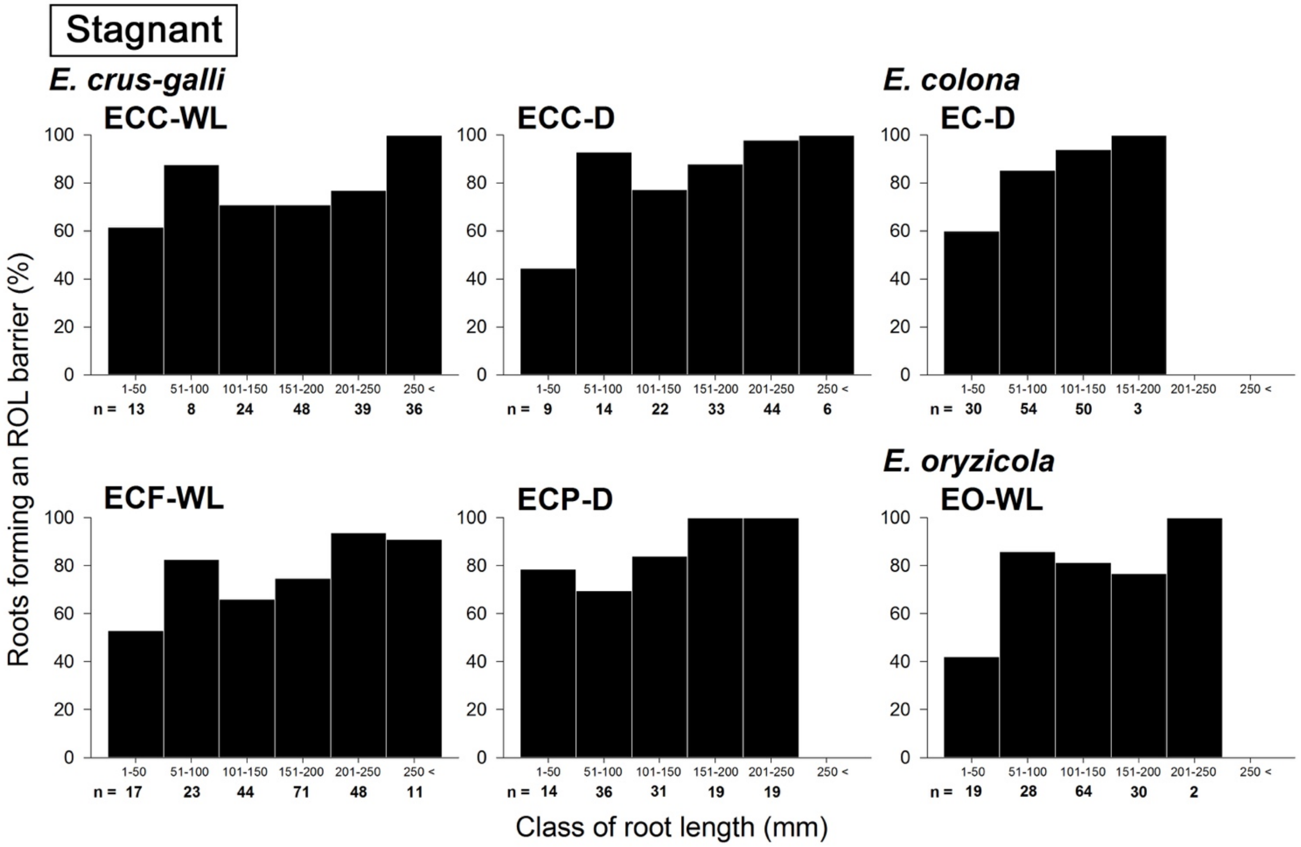

Supplementary Figure 4| Distributions of root length of adventitious roots with an ROL barrier in *Echinochloa* accessions under stagnant conditions. The data in this figure are replotted from the data in Figure 1 based on root length. All roots in 5 or 6 plants were used for each accession. Methylene blue was used to evaluate the formation of an ROL barrier in roots. Plants were grown in aerated nutrient solution for 10 days, and transferred to deoxygenated stagnant 0.1% agar solution for 14 days.

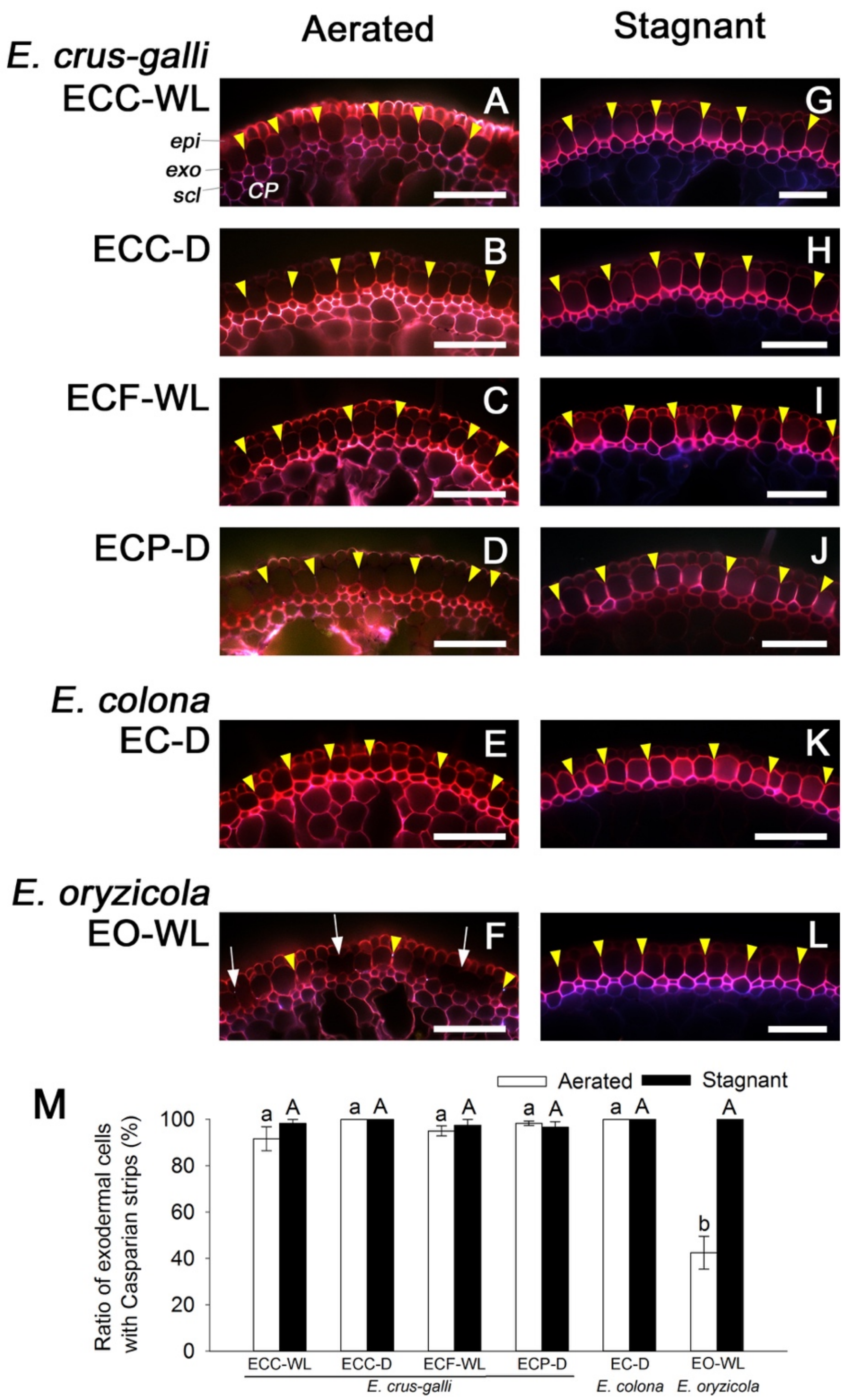

Supplementary Figure 5| Casparian strips at the exodermis in *Echinochloa* accessions that were grown under aerated or stagnant conditions. Casparian strips (stained by neutral red) were observed in the basal parts (15-25 mm below root-shoot junction) of adventitious roots of 100-120 mm length. **(A-L)** Casparian strips at the exodermis. Casparian strips are indicated as a bright red

fluorescence with neutral red. Representative Casparian strips are shown with yellow arrowheads. White arrows denote the region of exodermis/hypodermis without Casparian strips. Red fluorescence indicates autofluorescence. Abbreviations: *CP*, cortical parenchyma; *epi*, epidermis; *exo*, exodermis; *scl*, sclerenchyma. Scale bars, 100 µm. **(M)** Ratios of cell numbers observed Casparian strips. Means ± SE. $n = 4$. Different lower-case letters denote significant differences among *Echinochloa* accessions ($P < 0.05$, Fisher's exact test for multiple comparisons). Plants were grown in aerated nutrient solution for 10 days, and then transferred to deoxygenated stagnant 0.1% agar solution or continued in aerated solution for 14 days. Procedure of the Casparian strips staining using 0.1% (w/v) neutral red is described in **Supplementary Method 1**.

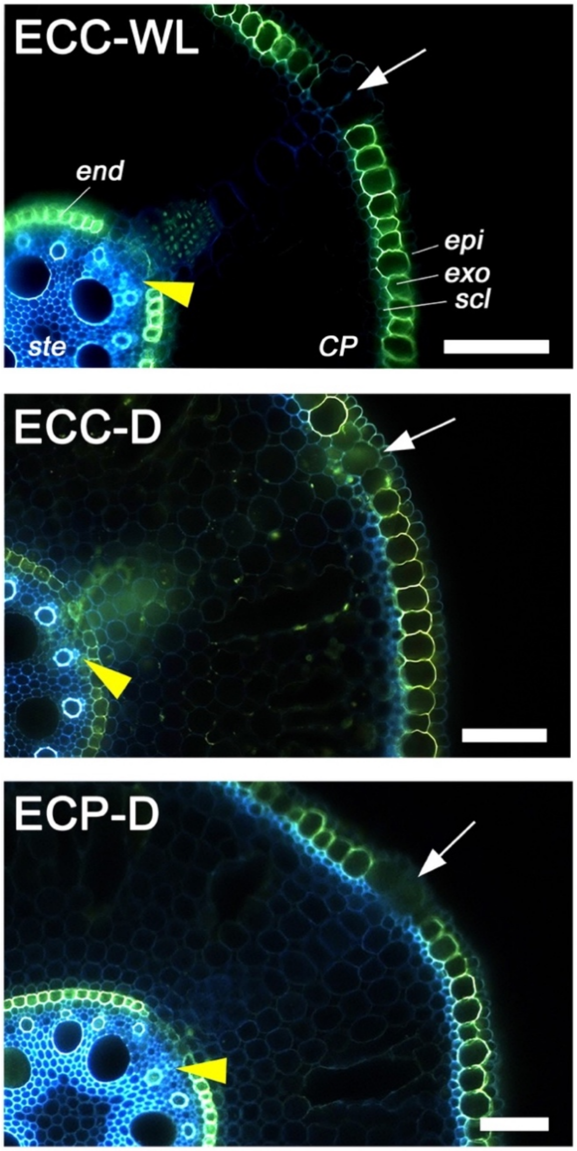

Supplementary Figure 6| Areas of passage cells (also called 'window') lack suberin lamellae at the exodermis emerging site of lateral roots in three *Echinochloa* accessions grown under aerated conditions. Suberin lamellae were visualized by Fluorol Yellow 088 at the basal parts (15-25 mm below root-shoot junction) of adventitious roots. White arrows indicate areas of passage cells (window) that lack suberin lamellae. Yellow arrowheads indicate the pericycle from which lateral root primordia are predicted to emerge. Blue fluorescence indicates autofluorescence. Plants were grown in aerated nutrient solution for 24 days. Abbreviations: *CP*, cortical parenchyma; *end*, endodermis; *epi*, epidermis; *exo*, exodermis; *scl*, sclerenchyma; *ste*, stele. Scale bars, 100 µm.

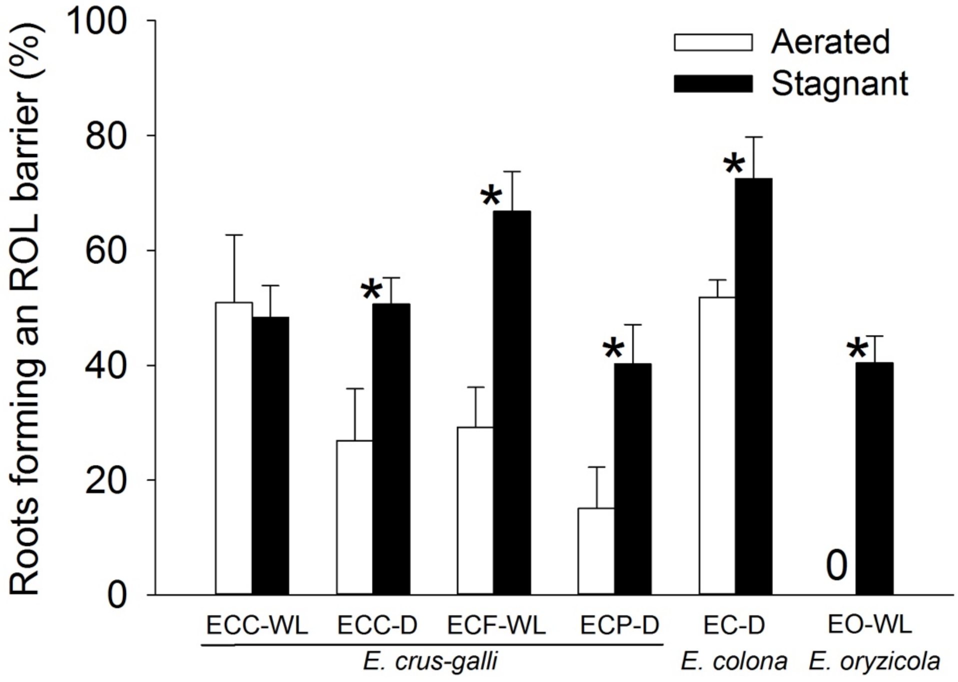

Supplementary Figure 7| Percentages of the roots that formed an ROL barrier in *Echinochloa* accessions grown under aerated or stagnant conditions for 7 days. Methylene blue was used to evaluate the formation of ROL barrier in roots. Means ± SE. n = 3 or 4. Asterisks denote a significant difference between aerated and stagnant conditions ($P < 0.05$, Fisher's exact test). Plants were grown in aerated nutrient solution for 10 days, and then transferred to deoxygenated stagnant 0.1% agar solution or continued in aerated nutrient solution for 7 days.

Supplementary Method 1. Visualization of Casparian strips with Neutral red

Adventitious roots (100-120 mm length) were cut at the root-shoot junction. Their basal parts (15-25 mm below root-shoot junction) were embedded in 5% (w/v) agar. Root cross-sections of ca. 100 µm thickness were made using a vibrating microtome (Leica VT1200S, Leica Biosystems). The cross-sections were made transparent by incubating them in lactic acid saturated with chloral hydrate at 70°C for 60 min (Lux et al., 2005). Casparian strips in the basal parts were stained with 0.1% (w/v) neutral red in 0.1 M phosphate buffer (pH 6.5) as previously described (Lulai and Morgan, 1992; Abiko et al., 2012), which appears as bright red fluorescence under UV light. The cross-sections were viewed with an 02 UV filter set, an Axio Imager.A2 and an AxioCam MRc CCD camera (Carl Zeiss). The ratio of cells with Casparian strips was determined by manually counting the numbers of cells in photograph. To reduce bias, we randomly selected 30 cells in a cross-section derived from four independent roots.

References

Lulai, E. C., and Morgan, W. C. (1992). Histochemical probing of potato periderm with neutral red: a sensitive cytofluorochrome for the hydrophobic domain of suberin. *Biotechnol. Histochem*. 67, 185-195. doi: 10.3109/10520299209110065
